# Complementary Food Ingredients Alter Infant Gut Microbiome Composition and Metabolism In Vitro

**DOI:** 10.3390/microorganisms9102089

**Published:** 2021-10-03

**Authors:** Shanthi G. Parkar, Doug I. Rosendale, Halina M. Stoklosinski, Carel M. H. Jobsis, Duncan I. Hedderley, Pramod Gopal

**Affiliations:** The New Zealand Institute for Plant and Food Research Limited, Private Bag 11600, Palmerston North 4442, New Zealand; doug.rosendale@anagenix.com (D.I.R.); halina.stoklosinski@plantandfood.co.nz (H.M.S.); carel.jobsis@plantandfood.co.nz (C.M.H.J.); duncan.hedderley@plantandfood.co.nz (D.I.H.)

**Keywords:** infant complementary foods, baby foods, gut microbiome, infant complementary feeding, infant solid foods, short-chain fatty acids, SCFAs

## Abstract

We examined the prebiotic potential of 32 food ingredients on the developing infant microbiome using an in vitro gastroileal digestion and colonic fermentation model. There were significant changes in the concentrations of short-chain fatty-acid metabolites, confirming the potential of the tested ingredients to stimulate bacterial metabolism. The 16S rRNA gene sequencing for a subset of the ingredients revealed significant increases in the relative abundances of the lactate- and acetate-producing Bifidobacteriaceae, Enterococcaceae, and Lactobacillaceae, and lactate- and acetate-utilizing Prevotellaceae, Lachnospiraceae, and Veillonellaceae. Selective changes in specific bacterial groups were observed. Infant whole-milk powder and an oat flour enhanced Bifidobacteriaceae and lactic acid bacteria. A New Zealand-origin spinach powder enhanced Prevotellaceae and Lachnospiraceae, while fruit and vegetable powders increased a mixed consortium of beneficial gut microbiota. All food ingredients demonstrated a consistent decrease in *Clostridium perfringens*, with this organism being increased in the carbohydrate-free water control. While further studies are required, this study demonstrates that the selected food ingredients can modulate the infant gut microbiome composition and metabolism in vitro. This approach provides an opportunity to design nutrient-rich complementary foods that fulfil infants’ growth needs and support the maturation of the infant gut microbiome.

## 1. Introduction

The trajectory of microbiome development in the first 1000 days of the child’s life [1,2] has long-term implications in terms of the individual’s immune and metabolic health [3]. A dysbiosis in the early-life gut and the interlinked alterations in the immune signaling have been associated with childhood immune-mediated disorders such as type 1 diabetes, juvenile asthma, and allergies [4,5].

Early feeding patterns, such as breast milk and/or formula and the duration of breastfeeding are some of the key factors in regulating gut bacterial colonization and composition and the associated immunological maturation of the growing infant [1,4]. Bifidobacteriaceae are the most abundant group in the gut of both breast-fed and formula-fed infants [2,6,7]. Seen in lesser abundance are bacterial families such as Bacteroidaceae, Lachnospiraceae, Veillenollaceae, Clostridiaceae, Lactobacillaceae, Enterococcaceae, and Streptococcaceae [2,6,7]. Formula-fed infants show a greater diversity in their gut bacteria with a higher abundance of families from the Firmicutes phylum [7,8]. Lactate producers such as Bifidobacteriaceae, Lactobacillaceae, Enterococcaceae, and Streptococcaceae have been shown to protect infants against antibiotic- and enteropathogen-induced diarrhea [9], inflammatory responses [10], and atopic dermatitis [11]. Bifidobacteria possess the metabolic capacity to degrade and utilize gut epithelial mucin and the structurally analogous human milk oligosaccharides [12,13]. Consequently, Bifidobacteriaceae have evolved to occupy prime niches in the infant gut [12,13] and may persist into adulthood [14,15]. A founder–colonizer species of the infant gut, *B. bifidum* lays the foundation for the subsequent colonization by diverse microbiota. This is enabled by sharing carbohydrate resources with other bifidobacteria, as well as intermediary metabolites such as lactate and acetate with butyrate- and propionate-producing bacteria such as *Eubacterium hallii* [12,16]. *B. bifidum* strains have also been associated with beneficial effects on gut functionality such as intestinal homeostasis, modulation of gut immunity, and anti-inflammatory protection [17,18]. Similarly, other bifidobacteria and lactic acid bacteria (LAB) have also been found to be autochthonous and remain gut-associated throughout the host’s lifespan, conferring beneficial effects on the host [14,19].

The transition from the milk-based infant diet to a mixed diet, where milk is complemented with plant-based foods, meat, and dairy, is recommended to start at around 6 months of age [1]. The period, up until 24 months of age, is a critical window of opportunity for shaping the structure of the developing infant’s gut microbiota [1]. Exposure to new foods triggers developmental maturation of the gut, and it presents novel, nondigestible carbohydrates to the microbes in the colon of the growing infant. Nondigestible carbohydrates have a major impact on the proliferation of gut bacteria such as *Bacteroides* and *Faecalibacterium* (the most abundant genera in Bacteroidetes and Firmicutes phyla, respectively, in the adult gut), and the concomitant reduction in enterobacteria, bifidobacteria, and clostridia that predominate in the infant gut [3,5,20]. The cessation from milk as the primary food and the transition to solid foods play an important role in the acquisition and proliferation of new bacteria and lay the foundation for a stable and adult-type microbial population. Foods that influence this early colonization may, thus, permanently shape gut microbiota composition, maturation of the gut and immune system, and even long-term health effects [1,5,21].

A variety of foods are being gradually introduced to growing infants as they adapt to the family’s dietary patterns. These early foods generally include cheese, meat, eggs, fruits, vegetables, and cereals, which are customized to local availability and family preferences [1,22,23]. As the food transits the growing child’s gastrointestinal tract, the fibers (and other phytochemicals such as polyphenols) that are resistant to host digestion reach the colon partially or fully undigested. The founding bacteria of the child, including bifidobacteria and LAB, adapt to the newly introduced dietary components, including fiber, which is broken down to generate lactate, an organic acid, and acetate, a short-chain fatty acid (SCFA) [4,5,14,24]. These microbial acid metabolites play an important role in the maturation of gut and gut ecology, including the development of syntrophic microbiota, by providing substrates to enhance the growth of secondary consortium that can deconstruct more complex carbohydrates to generate SCFAs such as propionate and butyrate. Lactate and the SCFAs also regulate microbial homeostasis by maintaining an acidic milieu that inhibits colonization by pathogens [25,26]. The SCFAs, mainly acetate, propionate, and butyrate, are also important for host health, with butyrate being particularly important as an energy source for colonocytes and for strengthening of gut barrier function [26,27]. Butyrate is generated mainly by Lachnospiraceae and Ruminococcaceae, either by breaking down polysaccharides or by utilizing the lactate produced by lactic acid bacteria and the acetate, an early metabolite of microbial metabolism of glycans [26]. The gut microbiome, especially when highly diverse and abundant in beneficial members of *Bifidobacterium*, *Bacteroides*, Lachnospiraceae, and Ruminococcaceae, can aid in the prevention of gut disorders, as well as autoimmune and allergic disease, during childhood and later in life [4,5,28,29].

Prebiotics are recognized as some of the most promising dietary supplements, with numerous health benefits in both children and adults. One of the most studied prebiotics in pediatric nutrition (infant formulae) is a mixture of short-chain galactooligosaccharide and long-chain fructooligosaccharide in a ratio of 9:1 [30]. Solid foods supplemented with galacto- and fructooligosaccharides were found to enhance gut bifidobacteria when fed in a 6 week intervention study involving 4–6 month old infants who were about to start consuming solid foods [31]. Inulin is another well-characterized prebiotic that has been shown to beneficially affect the gut microbiome of children [32,33] and is often used as a supplement in many food products formulated for children. Looking beyond galactooligosaccharides and fructans, exposure of the early-life gut microbiome to a wide array of nondigestible carbohydrates with varied physicochemical complexities helps to further enhance the plasticity of the microbiome [1,21]. Before progression to family foods, and to complement breast and/or formula feeding, infants are given suitable homemade or commercial ready-to-serve foods. Infant complementary foods comprise plant-based, dairy, and meat products and aim to meet the nutritional needs of infants [34,35,36,37].

Plant-based foods are a rich source of structurally varied carbohydrates, with varying capacities to alter gut microbial composition [1,38,39,40]. While different fractions of plant-derived carbohydrates are recognized to play an important role in shaping the infant gut microbiome, few studies have examined the microbiome-modulating role of whole foods or ingredients that may be used in the formulation of infant foods. We hypothesized that food ingredients (predigested in an upper gut model) will differentially modulate colonic microbiota from infants in a simulated hind gut model. In this study, we selected a range of commercially available plant-based food ingredients, along with whole-milk powders, and evaluated their ability to modulate infant gut microbiota using a batch fermentation model of the colon. We examined these ingredients for their fermentative capacity by following the changes in the metabolites generated by microbial breakdown of the substrates over 24 h, and we further studied a subset of the selected food ingredients in terms of changes in substrate-driven microbiome structure.

## 2. Materials and Methods

**Figure 1:**
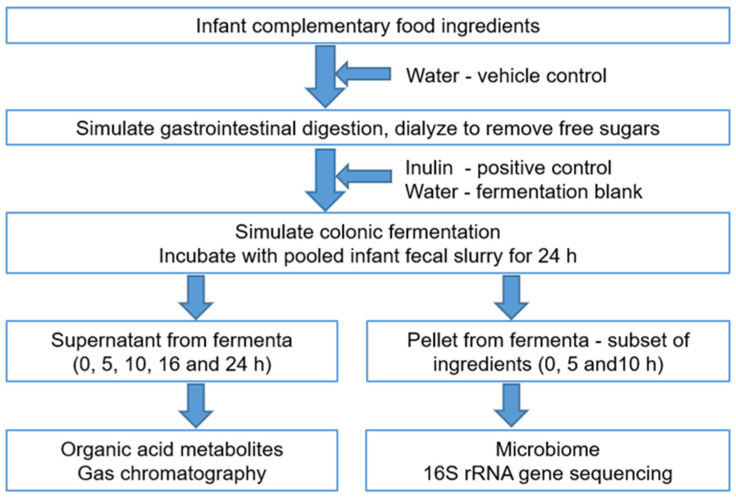
An outline of the experimental protocol and analyses.

A schematic workflow of the experimental protocol used in this study is shown in Figure 1.

### 2.1. Food Ingredients

Thirty-two different food ingredients were selected from commercial sources and tested for their ability to modulate infant gut microbiota. They were classified as per the United States Department of Agriculture [41] into milk, fruit (blackcurrant, Boysenberry, blueberry, kiwifruit, apple, feijoa, and passionfruit), vegetables (carrot, pea, pumpkin, spinach, sweetcorn, sweet potato, and tomato), flavor agents (honey and kaffir lime leaf), and different forms of cereal grain (oats) (Table 1). Orafti^®^ Synergy1 (ORAFTI Active Food Ingredients, Tienen, Belgium, hereafter referred to as inulin) was included as a positive control. This inulin is a 1:1 mixture of short-chain oligofructans and long-chain fructans that vary in their degrees of polymerization, i.e., <10 and 2–60, respectively. All the ingredients used were procured in dried powder formats.

### 2.2. In Vitro Simulated Gastroileal Digestion

As outlined in Figure 1, food ingredients were digested in vitro using previously published protocols [42]. Each food ingredient (*n* = 32) was weighed in triplicate (25 g) and hydrated in 46 mL of sterile de-ionized water. Three replicates of sterile deionized water (25 mL) were included as the vehicle control. The hydrated food ingredients and the water control were incubated with acidified 10% pepsin (P7000, >250 units/mL, Sigma-Aldrich, St. Louis, MO, USA) with slow constant stirring (130 rpm) at 37 °C for 30 min. The reaction mixture was buffered to pH 6.0 with 0.1 M maleate buffer and incubated with 0.05 mL of amyloglucosidase (E-AMGDF, Megazyme, Bray, Ireland) and 1.25 mL of 2.5% pancreatin (P7545; 8 × USP specifications, Sigma, St. Louis, MO, USA) at 37 °C for 120 min in a final volume of 27.5 mL. In a simulation of intestinal passive absorption of small molecules such as glucose, the reaction mixture (representing the digesta) was dialyzed using a 1000 Da molecular weight cutoff Spectra/Por^®^ CE membrane (Thermo Fisher Scientific, Auckland, New Zealand) with at least six changes of cold deionized water over 24 h. Additionally, three extra replicates of water were similarly prepared, and inulin (2.5 g) was added to the predigested water in a final volume of 27.5 mL. Inulin is resistant to host digestive enzymes [32] and is, therefore, not expected to change after digestion. Hence, inulin was added after the digestion and dialysis steps to avoid the loss of small-molecular-weight fructooligosaccharides during dialysis.

### 2.3. Preparation of Fecal Inoculum

Fresh feces were obtained from 14 healthy infants (aged between 5 and 12 months) with informed consent from their primary care givers. Approval #16/NTA/154, dated 7 October 2016, was obtained from the Health and Disability Ethics Committees, Ministry of Health, New Zealand. Relevant details of the infant donors who donated feces as a source of gut microbiota are provided as a supplementary table (Table S1).

The nappy liners containing fresh feces were transferred to gastight bags containing one Anaeropouch™ (Mitsubishi Gas Chemical Company Inc., Tokyo, Japan) and transported in an insulated lunch bag containing an icepack to the laboratory. The feces were processed within 1 h of defecation into a 25% *v*/*v* fecal slurry by homogenizing with chilled sterile pre-reduced glycerol in phosphate-buffered saline with 0.05% *w*/*v* cysteine, and aliquots were stored at −80 °C. All processing of feces was carried out anaerobically under an atmosphere of CO_2_:H_2_:N_2_ at 5:5:90 in a Coy anaerobic chamber (Coy Laboratory Products Inc., Grass Lake, MI, USA). Two hours before the fermentation, one aliquot of each of 14 fecal slurries was removed from −80 °C, thawed in the anaerobic chamber, and pooled in equal proportions for immediate use as the inoculum.

### 2.4. Simulated Colonic Fermentation

First, 3 mL of a 10× sterile pre-reduced carbohydrate-free basal medium, prepared as described previously [42], was added to each digesta (resulting from gastroileal digestion, as described in Section 2.3). The pooled fecal slurry was then added to the reaction mixture at a final concentration of 1% *v*/*v*. The final concentration of the food ingredients and inulin was 2.5 g in a final fermentation volume of 30 mL.

In addition to the water control, a fermentation blank, containing sterile deionized water with no digesta but incubated with fecal slurry, was included in triplicate. Thus, one set of 32 food ingredients, inulin, water, and the fermentation blank were fermented with freshly prepared pooled fecal inoculum at 37 °C for up to 24 h on each of three separate days (Figure 1). The fermentation blank was included to examine changes in microbiota-generated acid metabolites, as an indication of fermentative changes with “undigested” water. Two 1 mL aliquots were collected from the fermentation mixture at 0, 5, 10, 16, and 24 h, immediately centrifuged at 13,000× *g* for 5 min at 4 °C, and the pellets and supernatants were separated and stored at −80 °C until further processing. The supernatants were used for analysis of organic acids, while the pellets were used for extraction of DNA and subsequent microbiome compositional analysis.

### 2.5. Analysis of Organic Acid Metabolites

The concentrations of the organic acids, formate, acetate, propionate, isobutyrate, butyrate, isovalerate, valerate, hexanoate, heptanoate, lactate, and succinate, were quantified using gas chromatography as described previously [43], and data were measured as µmol/mL fermenta.

### 2.6. Characterization of Microbial DNA

DNA was extracted from 0, 5, and 10 h fermenta of the substrates, i.e., water, inulin, and a subset of the food ingredients, employing the Qiagen DNeasy PowerLyzer PowerSoil Kit (Bio-Strategy Ltd., Auckland, New Zealand), with some modification. Briefly, the bacterial pellets were shaken in the PowerBead tubes at 55 m/s for 60 s using a FastPrep-24™ 5G (MP Biomedicals, Solon, OH, USA) and cooled on ice for 5 min before further processing according to the manufacturer’s instructions. The extracted DNA was stored at −80 °C until use, and its quantity and quality were measured using Qiagen QIAxpert (Bio-Strategy Ltd., Auckland, New Zealand).

The V3–V4 region of the 16S rRNA gene was sequenced using an Illumina MiSeq 2× 250 base paired-end run [44]. The sequence data were analyzed using Quantitative Insights Into Microbial Ecology 2 (QIIME 2, v 2018.8) [45] using the DADA2 method for denoising and inferring the amplicon sequence variants (ASVs) [46]. The taxonomical identity of the ASVs was assigned using Greengenes database (v.13.8, with 99% sequence similarity) trained on the naïve Bayes classifier [47]. Diversity analysis was performed with unfiltered ASVs, with rarefaction at 21,000 reads using the QIIME2 pipeline. Microbial α-diversity examining variety and abundance of species within a sample was measured using Shannon index, richness, evenness, and phylogenetic diversity. Microbial β-diversity to examine similarities/differences in microbial communities between samples was measured in terms of Bray–Curtis index (dissimilarities in microbial abundance), unweighted uniFrac (measures community membership, as it records absence and presence of different ASVs), and weighted uniFrac (measures community structure, by accounting for the relative abundance of each ASV). Microbiota data ordination was done using a principal coordinates analysis (PCoA) plot based on the weighted uniFrac metric within the QIIME2 workflow and visualized as EMPeror plot [45].

### 2.7. Statistical Analyses

For the organic acid data, analysis of variance was used, with substrate (i.e., food ingredients, inulin or water control, and fermentation blank) and time as factors, and the replicate as a block (random). The data were log-transformed to stabilize the variance. Multiple comparisons between substrate means were performed using Tukey’s honestly significant difference (HSD).

The ASV dataset was filtered to include reads which were present at >0.05% relative abundance in at least one sample prior to differential abundance analysis using DESeq2 [48]. Combining DESeq2 with likelihood ratio tests and a nested factorial structure allowed testing for differences between the treatments at each timepoint, and then between the averages for each timepoint. The *p*-values were adjusted for false discovery rate.

The significant changes in terms of microbial α-diversity measures were calculated using the Kruskal–Wallis test. The significances in the β-diversity measures were calculated using the one-way permutational multivariate analysis of variance (PERMANOVA) pseudo-F method [45]. Differences were considered significant at *p* < 0.05.

Spearman’s rank correlation test was used to analyze correlations between organic acid concentrations and microbiome composition. Spearman’s correlations (*r*) significant at a false discovery rate-adjusted *p* < 0.05 are quoted.

## 3. Results

The organic acid profile was generated after 0, 5, 10, 16, and 24 h of fermentation of the food ingredients, inulin, water control, and the fermentation blank. Formate, acetate, propionate, lactate, and succinate were present in all samples (Table S2). They showed significant substrate, time, and substrate × time interactions, with the biggest effects being changes over time, along with differences between the mean values of the various samples (Table S3). Many of the substrates showed significant changes in the organic acids at 0 h. During the course of the fermentation, formate concentrations were low and steady for most substrates, while the values started high and declined for oat flour (F00151). Early formate peaks were seen for kaffir lime leaf (5 h) and spinach leaf (10 h). Acetate increased over time with all the substrates, with the increases being higher for the two milk powders and spinach. Lactate, like acetate, increased over time with all substrates, with increases being high for three oat flours (F00005, F00151, and Export) and low for spinach, water, and the fermentation blank. Propionate also increased for all the substrates, but more so with spinach and kaffir lime leaf. Butyrate increased only in milk powders, spinach, kaffir lime leaf powder, water, and the fermentation blank. Valerate increases were seen only with spinach, kaffir lime leaf, water, and the fermentation blank. Hexanoate increased with the milk powders. Heptanoate concentrations were below the limit of detection (Table S4). Isobutyrate and isovalerate were detected only in the water control and the fermentation blank from 10 h through 24 h.

**Figure 2:**
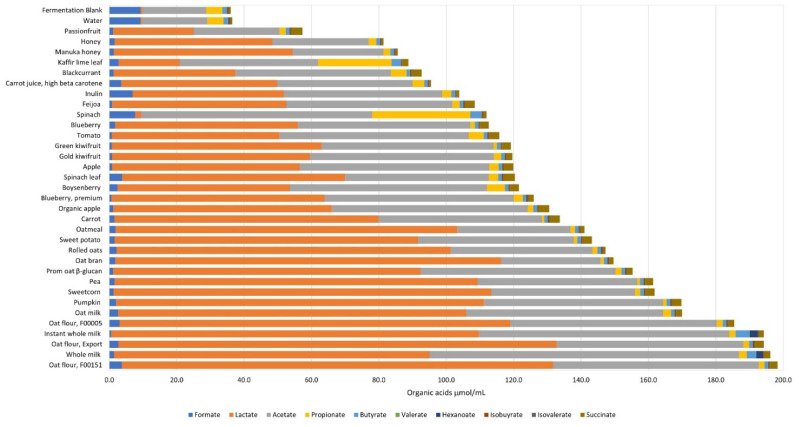
Average organic acid profiles of the 32 food ingredients, inulin (positive control), water, and the fermentation blank at 10 h fermentation with the pooled infant fecal slurry (*n* = 3). The statistical differences are given in Supplementary Tables S2 and S3.

At the end of 10 h (Figure 2 and Table S2), there were no significant changes (*p* < 0.05) in valerate, heptanoate or the branched-chain fatty acids, isobutyrate and isovalerate. In case of the ingredients, formate concentrations were the lowest with instant whole milk and the highest with spinach powder. Lactate concentrations were lowest with spinach and highest with the export type of oat flour. Acetate concentrations at 10 h were lowest in passionfruit powder and highest in whole-milk powder. Propionate was lowest in carrot and highest in spinach. Butyrate remained at 1 µmol/mL fermenta for most of the ingredients and was increased ~3-fold with kaffir lime leaf, spinach, and whole milk powder, and ~4-fold with instant whole milk powder. The 10 h inulin fermenta were rich in lactate and acetate, while water and the fermentation blank acid profiles were similar—low in lactate and acetate, and high in formate and propionate.

The microbiome characterized for the 0, 5, and 10 h fermenta from a subset of ingredients, inulin, and water revealed that a total of 7,036,016 reads were obtained, with the minimum number of reads per sample being 21,766. In the case of the 0 h sample, which is representative of the pooled fecal inoculum, the four major phyla present were Firmicutes, Proteobacteria, Actinobacteria, and Bacteroidetes, with their relative abundances (RAs) being 40%, 24%, 20%, and 16% respectively (Table S5a). The most abundant families that were over 10% RA were Enterobacteriaceae, Bifidobacteriaceae, Bacteroidaceae, and Veillonellaceae (Table S5b). Differential abundance analysis of the changes in the microbiome at 5 and 10 h fermentation revealed substrate-related effects on the microbiome at the phylum, family, and species levels or the closest classifiable taxonomical level (Tables S6–S8, respectively). At 10 h, significant substrate-mediated effects (*p* < 0.005) were seen in Actinobacteria, Bacteroidetes and Cyanobacteria (Table S6). Spinach powder showed the least Actinobacteria (3% RA compared to 37% for instant milk powder) and highest Bacteroidetes (33% RA compared to 4% with tomato powder). Presence of plant-derived Cyanobacteria (resolved to an unclassified family of Streptophyta) [49] was increased to ≥1% RA with blackcurrant and Boysenberry.

**Figure 3:**
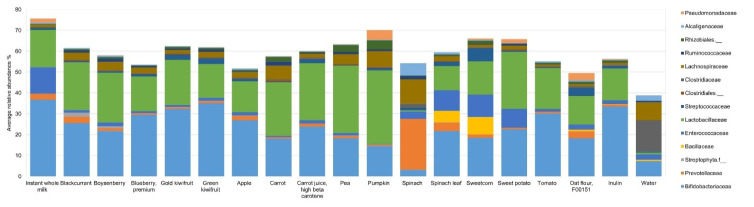
Average relative abundances (%) of bacterial families that differed significantly (*p* < 0.005) with food ingredients, inulin (positive control), and water at 10 h of fermentation.

At 10 h, several significant differences (*p* < 0.005) were observed at the family level (Table S7 and Figure 3). The relative abundance of Bifidobacteriaceae was the highest at 37% with instant milk powder and was between 15–35% for all other ingredients, except spinach powder (3%). Comparatively, spinach leaf powder increased the RA of Bifidobacteriaceae to 22%. Prevotellaceae was increased to 24% RA with spinach powder, while it was 4% RA with spinach leaf powder, with the values being between 0% and 3% RA for the remaining substrates. Bacillaceae was at 8% RA with sweetcorn powder, 5% RA with spinach leaf powder, and between 0% and 1% RA for the remaining substrates. Enterococcaceae relative abundance was highest with instant milk powder (13% RA) and lowest with pumpkin powder (0.25% RA), and it was generally low with fruit, carrot, pea, tomato, and oat powders, and high with spinach leaf, sweetcorn, and sweet potato powders. The RA of Lactobacillaceae (Lactobacillales) was highest at 35% with pumpkin powder, with the values being over 20% RA with some vegetable powders (i.e., pea, pumpkin, carrot, carrot juice, sweet potato) and fruit powders (blackcurrant, Boysenberry, gold-fleshed kiwifruit), with spinach powder far lower than the rest (0.6% RA). Streptococcaceae (also from the order Lactobacillales) was increased to 7% RA with sweetcorn powder compared with the smaller changes seen with the other ingredients. Lachnospiraceae relative abundance was high with spinach powder (12%), and to a smaller extent with pumpkin, carrot, and pea powders, at 8%, 7%, and 5% RA, respectively. Small but significant changes in relative abundance (<6%) were seen with some ingredients for Clostridiaceae, Ruminococcaceae, Alcaligenaceae, Pseudomonadaceae, and unclassified families of Streptophyta, Clostridiales, and Rhizobiales. The fermentation positive control, inulin, induced large increases in the relative abundances of Bifidobacteriaceae (33%) and Lactobacillaceae (15%). Water, the vehicle control, increased the relative abundances of Clostridiaceae (15%), Lachnospiraceae (9%), Enterococcaceae (3%), and Alcaligenaceae (3%).

While acknowledging a loss of resolution in ascribing genus- and species-level data with 16S rRNA gene sequencing [50], we studied the microbiome changes at the species level at 10 h and observed some significant changes, as shown in Table S8. In the case of Bifidobacteriaceae, significant changes were seen in *Bifidobacterium* spp. (*p* < 0.05), *B. bifidum* (*p* < 0.005), *B. adolescentis* (*p* < 0.005), and *B. longum* (*p* < 0.05). *B*. *bifidum* was increased highest by green-fleshed kiwifruit powder (12% RA) followed by tomato powder (10% RA). *B. longum* was at similar relative abundances for most ingredients, the greatest for instant milk and green-fleshed kiwifruit powders (14% RA), and the lowest for spinach powder (2% RA). An unclassified *Bifidobacterium* species was significantly higher (*p* = 0.01) for green-fleshed kiwifruit (13% RA) and spinach leaf powders (14% RA, in contrast to spinach powder, which was the lowest at 1% RA), while all the other ingredients were between 4% and 12% RA. Of the changes in the Bacteroidetes phylum, the most remarkable effect was the 30% RA in *Prevotella copri* with spinach powder (*p* < 0.005). Other changes where the RA of the species was at least 5% with at least one substrate were *Bacteroides* spp. (*p* < 0.05) and *B*. *ovatus* (*p* < 0.04). These species were at lower RA with instant whole milk, blueberry, carrot juice, sweet potato, and tomato powders. Carrot (in contrast to carrot juice, high β-carotene at 3% RA), apple, and oat flour, F00151 powder, among others, were stimulatory of *Bacteroides* spp. (9%, 7%, and 7% RA, respectively). The changes in Firmicutes were driven mainly by alteration in the classes Bacilli (*Bacillus* spp. And LAB) and Clostridia (families Clostridiaceae, Lachnospiraceae, Ruminococcaceae, and Veillonellaceae). *Bacillus* spp. Were less than 1% RA for all substrates, except sweetcorn and spinach leaf powders at 12% and 7% RA, respectively. Of the LAB, all the substrates, except spinach powder and water, increased at least one of the following genera by over 5% RA: *Enterococcus* spp., *Enterococcus* sp., *Lactobacillus* spp., *Lactobacillus zeae*, and *Weissella* spp. (all at *p* < 0.005) and *Lactobacillus reuteri* (*p* < 0.05). Some significant differences included the 9% RA in *Enterococcus* spp. And 5% RA in *Lactobacillus reuteri* with instant milk powder. The fruit powders (blackcurrant, Boysenberry, gold-fleshed and green-fleshed kiwifruit, and apple), vegetable powders (carrot juice, pea, and tomato) and inulin increased only *L. reuteri* as compared to the other LAB. The increase was greatest with carrot juice powder (19% RA) and lowest with inulin (7% RA). Carrot juice and pea powders caused a similar trend, except that *L. reuteri* values were 16% and 19%, respectively. Two different groups of LAB were increased to >5% RA with instant milk and sweet potato powders (*Enterococcus* spp. and *L. reuteri*), blueberry powder, and oat flour, F00151 (*L. reuteri* and *Weissella* spp.), carrot and pumpkin powders (*Lactobacillus* spp. and *L. reuteri*), and sweetcorn powder (*Enterococcus* sp. and *L. reuteri*). Spinach leaf powder modulated four different LAB groups to >5% RA, namely, *Enterococcus* spp., one unclassified *Enterococcus* sp., *L*. *reuteri*, and *Weissella* spp. Notable ingredient-driven effects on Clostridia with significant changes ≥5% RA in any one putative species were observed. The major changes in Lachnospiraceae were with spinach powder, which caused a bloom in at least two unidentified Lachnospiraceae species, and pumpkin powder, which increased *Blautia* spp. (5% RA). Compared with the other substrates, water increased *Clostridium perfringens* to 16% RA, and this was suppressed to <0.5% RA by all ingredients, except spinach powder (2% RA). Water also modulated *Phascolarctobacterium* spp. to 6% RA. Smaller but significant modulations (1% to 2% RA) in *Ruminococcus gnavus* and *Phascolarctobacterium* spp. were seen with most substrates. Another genus from Veillonellaceae, i.e., *Veillonella* spp., was also modulated (*p* < 0.05) by the substrates, with >5% RA being seen with the fruit powders, carrot juice powder, spinach powder, tomato powder, and water. Other small but significant (*p* < 0.005) increases were with two Proteobacteria, an unclassified group from Rhizobiales, and *Sutterella* spp. (7% RA by spinach powder).

The Spearman’s correlation coefficient for the 10 h organic acid and microbiome abundances revealed that *Bifidobacterium longum* correlated with formate (*r* = −0.731), *Veillonella* spp. with lactate (*r* = −0.774) and propionate (*r* = 0.819), and unclassified Acetobacteraceae species with propionate (*r* = −0.766). In the cases where both the acid and the organism were found in only one substrate, i.e., milk powder, a Spearman’s *r* of 1 was calculated. These were for *Pediococcus* with hexanoate and *Pseudomonas fragi* with hexanoate.

**Figure 4:**
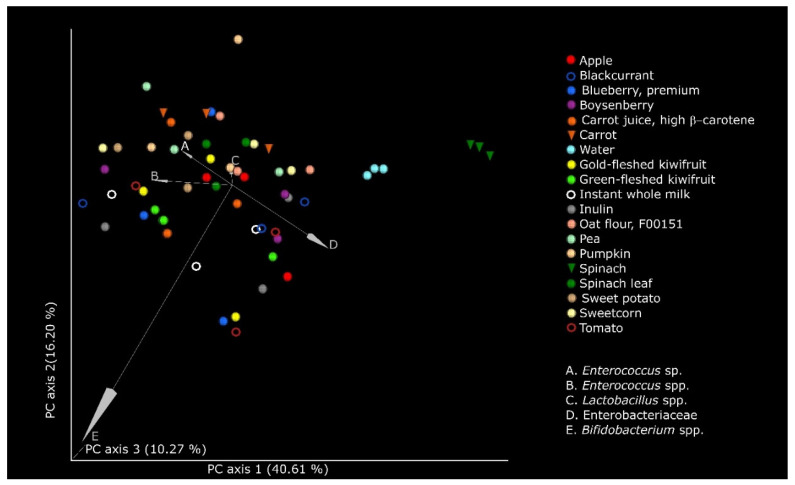
Principal coordinates analysis (PCoA) plots based on weighted UniFrac distances demonstrating significant shifts in the microbiome structure at 10 h of fermentation of the food ingredients, inulin (positive control), and water. Each sample was anaerobically fermented in triplicate, in the presence of pooled infant fecal slurry. The axes represent the dimensions explaining the greatest proportion of variances in the communities. The statistical significance (*p* < 0.005) was computed using permutational multivariate analysis of variance (PERMANOVA).

The changes in the microbiome after fermentation were examined at 5 and 10 h (separately) in terms of microbial diversity. There were no significant changes in terms of the within-community α-diversity at 5 or 10 h (data not presented). There were, however, significant changes (*p* < 0.005) in the β-diversity at both 5 and 10 h, with both nonphylogenetic (Bray–Curtis dissimilarity index) and phylogenetic (weighted and unweighted UniFrac) metrics. The EMPeror plot demonstrating the separation of the communities using the weighted uniFrac distances, which is a measure of the structure of the microbial community at 10 h of fermentation, is depicted in Figure 4. The taxa that most influenced the separation of the microbial communities between the different substrates were *Enterobacteriaceae*, *Lactobacillus* spp., *Enterococcus* spp., and *Bifidobacterium* spp. Community structure clusters (weighted UniFrac) were most clearly visualized for spinach and spinach leaf powders and water control.

## 4. Discussion

Thirty-two food ingredients were taken in equal quantities and investigated for their ability to modulate the gut microbiome of the growing infant using simulated in vitro models of gastroileal digestion, intestinal absorption, and colonic fermentation. Examining the changes in microbial organic acid metabolites and microbial composition, we inferred the fermentative potential of these ingredients with the infant gut microbiome.

Similar in vitro models have previously been used to study the fermentative capacity of foods and ingredients with infant gut microbiome and validated using clinically proven prebiotics, including fructo- and galactooligosaccharides as positive controls [32,51]. In our study, water was included as a control to confirm the potential effect of the digestive enzymes that may escape digestion and dialysis, thereby reaching the colonic fermentation stage. Infants up to the age of 9 months harbor very low numbers of groups such as the butyrate-producing Lachnospiraceae and Ruminococcaceae that are more indicative of microbial diversity and adaptation to different foods [2,4,52]. Therefore, to have a baseline that includes different microbiota that may potentially be modulated in this study, fecal bacteria obtained from infants 5–12 months of age were pooled to generate an inoculum with a diverse representation of bacteria that might be encountered by the infant. Thus, limitations of an in vitro system (vs. a clinical trial) and a fixed and sparse microbial baseline (vs. acquisition of new microbes) were mitigated by using appropriate standards and a pooled microbial inoculum, respectively.

Food ingredients selected were powders of different fruits, vegetables, varieties of oats, and milk, which are all often components of infant foods [1,34]. Powdered food ingredients have a stable shelf-life compared to fresh foods, are more consistent in terms of composition, are easier to transport, and are often used in baby food formulation. Commercially available sources were chosen to allow future use in the formulation of new infant complementary food with reproducibility of results. A standardized amount (2.5 g/30 mL) was chosen rather than a normalization with respect to the fiber content of such powders, as bioactives other than fiber are now being recognized as modulators of the gut microbiome. These bioactives include polyphenols that are present within the plant cell structures and potentially reach the colon [53,54].

The presence of organic acids in the baseline fermenta of many of the samples is indicative of the organic acids that were present in the substrate or generated during digestion, but not dialyzed out, despite their low molecular weight. While the fermenta were collected within 0.5 h, we do not consider that the fecal microbial metabolism was sufficiently activated to generate microbial acids. The increase in lactate (and acetate) to varying degrees throughout the fermentation may be attributed to the fact that most plant-based foods contain oligosaccharides that are rapidly metabolized. The exceptions, including spinach powder and lime leaf powder, which both showed increases in propionate and acetate, and to a smaller extent butyrate, indicated a syntrophic metabolism that comes into play when the early acid metabolites are further utilized by specialist bacteria. In the case of spinach powder, the early increase in succinate (at 5 h) depleted with the increase in propionate at 10 h. The smaller early rise in butyrate that built up over the course of the fermentation again indicates the increase in butyrogenic clostridia [26,55]. The accompanying increase in formate is a recognized mechanism to generate more acetate via mutualistic cross-feeding of hydrogen-utilizing acetogens, which then generate butyrate [56]. Most other ingredients caused lactate and acetate production to varying degrees, which is an indication of the microbial utilization of the carbohydrates that are still available in this closed system. The comparatively low propionate and butyrate production with infant fecal microbiota as compared to adult fecal microbiota indicates the immaturity of the microbiome and its inability to utilize carbohydrates efficiently [4,5,39,42]. In addition to the development stage of the infant microbiome, the fermentation rates and metabolic profiles of the different foods are influenced by the food components, especially the amounts and the physicochemical nature of the carbohydrates [21,57]. Indeed, fermentation is higher for linear polysaccharides (inulin, β-glucan, resistant starch) than for carbohydrates with sidechains such as arabinoxylans. This is consistent with the high lactate and acetate for the milk powders and certain vegetable powders (pea, pumpkin, sweet corn, and sweet potato, but not carrot, spinach, and tomato), as well as the oats.

For the first-pass step of fermentation followed by SCFA analysis, the variety of ingredients included different products from the same starting material, to determine the extent of variability that similar foods show in terms of their microbial stimulatory capacity. The variation in the SCFA profile indicates that the nature and the processing of the food are important factors. While honey is not usually recommended in foods for infants until the age of 1 year [58], we included it in this in vitro study to gather information on its prebiotic properties [43,59]. Most of the honey sugars were anticipated to be absorbed; the increase in microbial SCFAs indicates that the prebiotic mono- and oligosaccharides known to be present in honey may have been available to the fecal bacteria.

A subset of the ingredients that were anticipated to be more commonly used in infant foods was further analyzed for changes in microbiome composition. The timepoints of 5 and 10 h are close to the mean gastrointestinal transit time of 8.5 h (for 1–3 months old) to 10 h (for 1–2 years old) [60]. The inoculum obtained from infants may be considered representative of an in vivo system, while the batch fermentation employed in this study is a closed system precluding the replenishment of substrates or removal of bacterial metabolites and wastes. Furthermore, since pH was controlled only by buffering capacity of the bacterial fermentation medium, later timepoints may have confounding influences due to altered pH affecting microbial growth [61]. For these reasons, changes in the microbiome and its metabolites were compared within each timepoint. Acquisition was not simulated in this study, and this study, therefore, examines the ability of the ingredient–inoculum interactions to stimulate bacteria that are already present, but in very low relative abundances. The zero-hour composition analysis ascertained the presence of a diverse species of bacteria much like the adult gut microbiome. This implies an opportunity for the diverse bacteria to utilize the carbohydrate resources within ingredients to result in populational shifts in the microbiota. Indeed, changes in the community structure (weighted uniFrac) were driven by the different substrates.

Instant milk powder, which is often a component of infant complementary foods, is a source of lactose and milk oligosaccharides, both of which stimulate bifidobacteria, particularly *B. longum* subsp. *longum* in vitro [21,62]. Consistent with these in vitro results, when 7–90 day old infants were fed exclusively a cow’s milk-based formula or breast milk, an increase in bifidobacteria was observed at 3 weeks of feeding in both cases, although there was no difference in the concentration of SCFAs [63]. Interestingly only one, but not all the products tested behaved in a similar manner with respect to the effect on abundance of *Bifidobacterium* spp. This may have been due to differences in the composition of different products. *Bifidobacterium* spp. and *B. longum* were increased with the infant milk powder in this study. This suggests potential benefits due to its ability to persist in the gut beyond infancy and to metabolize a variety of dietary carbohydrates that are introduced during the complementary feeding [15,64]. Thus, higher bifidobacteria and LAB, and the associated lactate and acetate generation indicate microbial ecology driven by prebiotic components in the instant milk powder [62]. The greater lactate and acetate seen with foods such as sweet potato, sweetcorn, pea, and pumpkin powders, as well as oat flour, may be a consequence of the higher bifidobacteria, enterococci, and lactobacilli. This indicates a presence of easily metabolizable carbohydrates with fewer complexities or sidechains [65,66]. Carrots and fruits such as kiwifruit, apple, blackcurrant, and other berries are known to contain pectin-rich cell-wall polysaccharides [67,68]. Pectins enable the growth of a more diverse microbial consortium, led by the early blooms of bifidobacteria, lactobacilli, and streptococci [21,39,40,69]. The resultant lactate and/or acetate may have facilitated the growth of a second line of bacteria, e.g., Lachnospiraceae. While the increases in Firmicutes were not sufficient to cause large increases in butyrate, a prebiotic environment may be generated that supports beneficial propionigenic and butyrogenic commensals [6,70]. Spinach and tomato have a cell wall mainly composed of cellulose, hemicellulose, and lignin, and these components are known to be comparatively slowly fermentable by human gut bacteria [71,72]. This explains the lower lactate and acetate, and higher propionate and butyrate for these substrates, as some members utilize the easily accessible sugars, while other members further metabolize the primary end-products. Spinach cell-wall components also show phenolic cross-linkages, which makes it particularly recalcitrant to microbial breakdown [71]. This has a beneficial role, as the undigested fiber is moved further to the normally carbohydrate-poor distal colon for further microbial utilization. The high *P. copri* seen here with spinach powder is known to drive an increase mostly in succinate [26,73]. The further conversion of succinate to propionate and butyrate by cross-feeders such as *Faecalibacterium prausnitzii* and *Phascolarctobacterium* spp. [74,75] was not clearly evident in this study owing to the microbial composition of the immature inoculum, with its lower Firmicutes relative abundance, compensated for by Proteobacteria and Actinobacteria.

The differential effects on the microbiome changes caused by powders sourced from similar cultivars, such as carrots and spinach, may be explained by the different processing conditions. Thus, the type of food processing and consequent impact on food structure and composition (fiber, polyphenol content) may potentially influence microbiome composition [54,57,76,77]. This may explain the increase in *Bacteroides* spp. and Lachnospiraceae with the fiber-rich carrot powder made from whole carrots, but not the fiber-free carrot juice powder which was high in β-carotene. Similarly, spinach powder sourced from New Zealand *Spinacia oleracea* enhanced propionate (and butyrate), with the largest increase in *P. copri*. This propionigenic effect was not observed with spinach leaf powder, which had a similar macronutrient composition but “was sourced from imported spinach” according to the manufacturer’s product description.

Exposure of infants and toddlers to a variety of foods helps to build a more versatile gut microbiome. This study, along with other studies, demonstrates that foods differentially modulate infant gut microbiota [39,51]. Blending different food groups may help to improve the food palatability [78], as well as the nutritive content and variety [79], and generate combinations that provide age-specific support to the developing infant’s gut microbiome [28,52].

The inulin (positive control) was a commercial mixture of low- and high-molecular-weight oligofructans and was added to aliquots of dialyzed water digesta to avoid removal by the 1000 Da molecular weight cutoff dialysis membrane. The strong bifidogenic effect of inulin, as expected [32], validated our in vitro gut model. The water control was essentially a mixture of undialyzed digestive enzymes fermented with infant fecal bacteria in a peptone-rich broth. It served as a vehicle control in this study, but also mimicked a gut environment that was devoid of carbohydrate, but rich in proteins. The increase in *C. perfringens*, along with smaller increases in other Firmicutes families and *Bacteroides* spp., supports a metabolomic profile rich in propionate, butyrate, and branched-chain fatty acids such as isobutyrate and isovalerate, characteristic of the degradation of glycoproteins or proteins that reach the distal gut [26,80]. The ingredients were able to suppress *C*. *perfringens* [81], a potential pathogen, and this may be attributed to the increase in other beneficial bacteria. Spinach powder, which increased isobutyrate and isovalerate, still suppressed *C*. *perfringens*, while favoring the butyrate-producing Lachnospiraceae.

## 5. Conclusions

Our results show that powdered food ingredients displayed varied abilities to stimulate microbial metabolism, evidenced by the generation of beneficial SCFAs. The foods also enhanced desirable bacteria such as *Bifidobacterium*, *Bacteroides*, *Prevotella,* LAB, and Lachnospiraceae. Different foods were shown to selectively enhance specific groups; for example, infant whole-milk powder and oat flour, F00151, enhanced Bifidobacteriaceae and LAB, and spinach powder enhanced Prevotellaceae and Lachnospiraceae, while fruit and vegetable powders modulated a mixed consortium of beneficial bacteria. In addition, all the food ingredients were consistent in inhibiting the opportunistic pathobiont, *C. perfringens*, which was high only in the carbohydrate-free water control. More studies examining these food ingredients and their appropriate dosages should be undertaken to understand how different ingredients interact with the infant microbiome. This will help us to design nutrient-rich foods suited to the developmental stage of the infant.

## Figures and Tables

**Table 1:** List of ingredients subjected to in vitro gastroileal digestion and colonic fermentation. The fermenta were analyzed for organic acid metabolites at 0, 5, 10, 16, and 24 h of fermentation. The fermenta from a subset of ingredients (marked with an asterisk) were also analyzed for microbiota composition using 16S rRNA gene sequencing at 0, 5, and 10 h of fermentation.

Ingredient	Brief Description	Source
Instant whole milk	Ingredients: whole milk (cow’s), skim milk (cow’s), lactose, lecithin, vitamins A and D3	Miraka Limited, Taupo, New Zealand
Whole milk	Ingredients: whole milk (cow’s), skim milk (cow’s), lactose	Miraka Limited, Taupo, New Zealand
Blackcurrant *	Freshly harvested blackcurrants, destrigged, freeze dried, milled	Sujon, Gibb Holdings (Nelson) Ltd., Nelson, New Zealand
Boysenberry *	Freshly harvested Boysenberries, freeze dried, milled	Sujon, Gibb Holdings (Nelson) Ltd., Nelson, New Zealand
Blueberry	Freshly harvested blueberries, freeze dried, milled	Sujon, Gibb Holdings (Nelson) Ltd., Nelson, New Zealand
Gold-fleshed kiwifruit *	Fruit freeze-dried, milled	Fresh As, Auckland, New Zealand
Apple *	’Braeburn’ apple, freeze-dried, milled; ascorbic acid added as preservative	Fresh As, Auckland, New Zealand
Feijoa	Fresh feijoa, freeze-dried, milled	Fresh As, Auckland, New Zealand
Passionfruit	Deseeded passionfruit pulp, freeze-dried, milled	Fresh As, Auckland, New Zealand
Carrot *	Fresh carrot, freeze-dried, milled	Fresh As, Auckland, New Zealand
Spinach*	NZ-grown spinach, freeze-dried, milled	Fresh As, Auckland, New Zealand
Kaffir lime leaf	Kaffir lime leaves, freeze-dried, milled	Fresh As, Auckland, New Zealand
Blueberry, premium *	Whole blueberries, freeze-dried, milled	NutraDry, Hendra, Australia
Green-fleshed kiwifruit *	Fruit puree, freeze-dried, milled	NutraDry, Hendra, Australia
Organic apple	Fruit puree, freeze-dried, milled	NutraDry, Hendra, Australia
Carrot juice, high beta-carotene *	100% carrot juice (minus fiber), freeze-dried, milled	NutraDry, Hendra, Australia
Spinach leaf *	Whole spinach leaves, pureed, freeze-dried, milled	NutraDry, Hendra, Australia
Sweet potato *	Whole orange-fleshed sweet potato, pureed, freeze-dried, milled	NutraDry, Hendra, Australia
Sweetcorn	Mature super sweet sweetcorn kernels, pureed, drum-dried, milled	Cedenco Foods New Zealand Ltd., Gisborne, New Zealand
Pea *	Peas pureed, drum-dried, milled	Cedenco Foods New Zealand Ltd., Gisborne, New Zealand
Pumpkin *	Pumpkin peeled, pureed, drum-dried, milled	Cedenco Foods New Zealand Ltd., Gisborne, New Zealand
Tomato *	Tomatoes pureed, concentrated, mixed with starch carrier, drum-dried and milled	Cedenco Foods New Zealand Ltd., Gisborne, New Zealand
Oat flour, F00151 *	Milled oat flour	Harraways, Dunedin, New Zealand
Oat flour, F00005	Finely milled oat flour	Harraways, Dunedin, New Zealand
Oat flour, Export	Low moisture and water activity	Harraways, Dunedin, New Zealand
Rolled oats	Starting material for oat flour	Harraways, Dunedin, New Zealand
Oatmeal	Coarser particles than oat flour	Harraways, Dunedin, New Zealand
Oat bran	Coarse particles, higher source of fiber than oats	Harraways, Dunedin, New Zealand
Oat milk	Dehydrated oat milk	Harraways, Dunedin, New Zealand
PromOat^®^ Beta Glucan	Fine oat powder with 35% β-glucan	Tate & Lyle ANZ Pty Ltd., Auckland, New Zealand
Honey	70% honeydew honey, 30% maltodextrin	G & S Foods, Canvastown, New Zealand
Mānuka honey	70% mānuka honey, 30% maltodextrin	G & S Foods, Canvastown, New Zealand

## Data Availability

The 16S rRNA gene sequence data and metadata were deposited into the SRA database with links to the BioProject accession number PRJNA669972 (https://www.ncbi.nlm.nih.gov/bioproject/).

## Supplementary Materials

The following are available online at https://www.mdpi.com/article/10.3390/microorganisms9102089/s1: Table S1. Details of the infants donating the feces; Table S2. Organic acid concentrations during the course of fermentation; Table S3. Analysis of variance in the organic acid measurements; Table S4. Limits of detection of organic acid analysis; Table S5. Percent relative abundance of microbial phyla at 0 h at phylum level (**a**), family level (**b**), and species level (**c**); Table S6. Percent relative abundance of microbial phyla 5 and 10 h after fermentation; Table S7. Percent relative abundance of microbial families 5 and 10 h after fermentation; Table S8. Percent relative abundance of microbial species 5 and 10 h after fermentation.
